# Evaluation of Lower Limit of Fatigue Strength in High Cycle and Very High Cycle Fatigue Regime

**DOI:** 10.3390/ma18245621

**Published:** 2025-12-15

**Authors:** Saisai Wang, Yajun Zhang, Yifei Gao, Chengqi Sun

**Affiliations:** 1 State Key Laboratory of Nonlinear Mechanics, Institute of Mechanics, Chinese Academy of Sciences, Beijing 100190, China; wangsaisai22@mails.ucas.ac.cn; 2 School of Engineering Science, University of Chinese Academy of Sciences, Beijing 100049, China; 3 National Key Laboratory of Marine Corrosion and Protection, Luoyang Ship Material Research Institute, Luoyang 471023, China; 4 NCS Testing Technology Co., Ltd., Beijing 100081, China; gaoyifei@ncschina.com

**Keywords:** very high cycle fatigue, fatigue strength, sample size, up-and-down method, continuous testing method

## Abstract

**Highlights:**

**What are the main findings?**

- The CTM gives a reliable evaluation of the lower limit of fatigue strength.
- The evaluated results of 10~14 samples are generally close to those of 15 samples.

**What is the implication of the main finding?**

- The CTM can be used to evaluate the lower limit of fatigue strength.
- A sample size of 10 is a choice for fatigue strength evaluation with the CTM.

**Abstract:**

In this paper, very high cycle fatigue (VHCF) strengths are first evaluated for the TC17 titanium alloy and the welding joint of the 6005A-T6 aluminum alloy using the up-and-down method (UDM) and continuous testing method (CTM). Then, the influence of the sample size on the lower limit of the high cycle and VHCF strength (10^7^, 10^8^ and 10^9^ cycles) is investigated based on the experimental data and the previous results for aluminum alloys, steels and titanium alloys. It indicates that, compared to the common size of 15 samples used for the UDM, a sample size of 10~14 can generally give an acceptable evaluation of the lower limit of fatigue strength (LLFS) at 90% and 95% survival probabilities (SPs) and 95% confidence for both the UDM and CTM. The absolute value of the relative difference value of the result of 10~14 samples compared to that of 15 samples is generally within 5%. In addition, the UDM could give very dangerous evaluated results, and it fails to evaluate the LLFS in some cases. The CTM deals with all the testing results and gives a safe evaluation of the LLFS. Ten samples and an LLFS at 90% SP and 95% confidence can be preferred for fatigue strength evaluations using the CTM in a high cycle and VHCF regime.

## 1. Introduction

Fatigue is a main failure mode for metallic components and parts. The factors influencing fatigue behavior are various, e.g., the microstructure [1,2], stress ratio [3,4], temperature [5,6], loading history [7], etc. In particular, the VHCF behavior of metallic materials has been widely investigated in recent years [8,9,10]. However, different from low cycle and high cycle fatigue, VHCF tests consume much more time. For example, testing 10^9^ cycles requires about 386 d at a frequency of 30 Hz and 116 d at a frequency of 100 Hz, respectively. Thus, the ultrasonic frequency (20 kHz) fatigue test is widely used for the study of VHCF behavior in order to promote testing efficiency [11,12]. However, VHCF strength testing, especially at 10^9^ cycles, is still rarely reported in the literature.

Fatigue life or fatigue strength can be greatly scattered, even for the same specimens tested under the same conditions. Therefore, statistical analysis is an effective method for evaluating fatigue life or fatigue strength in order to obtain more reliable results. Many studies have been carried out based on probabilistic and statistical theories [13,14,15,16,17]. For example, the up-and-down method (UDM) is one of the most popular methods for testing and evaluating the fatigue strength of metallic materials [18,19]. In this method, the mean and standard deviation of fatigue strength are first estimated at a given fatigue life, and then the first stress level and stress step are chosen. After that, the first sample is tested at the first stress level. If it fails before the given fatigue life, the tested stress level is decreased by a stress step for the next sample, or else the tested stress level is increased by a stress step for the next sample. The experiment is stopped until all the target samples are tested.

More recently, Wu et al. [20] proposed a continuous testing method (CTM) for evaluating the high cycle and VHCF strength based on probability and statistics theory. In this method, the first stress level and stress step *d* are selected, as in the UDM. Then, tests are conducted at lower or higher stress levels until three samples (the highest level, *S*_1_, fails, and the adjacent two lower stress levels, *S*_1_−*d* and *S*_1_−2*d*, run out) are obtained. The specimens are tested at *S*_1_−2*d* or/and *S*_1_−3*d* until all the target samples are finished. Different from the UDM, the samples can be tested simultaneously at different stress levels, i.e., the CTM is irrespective of the testing order of samples. Thus, it can greatly improve the testing efficiency, especially for the fatigue strength at a long fatigue life, e.g., it could reduce the testing period by over 66.7% for 16 samples compared to the UDM when the testing condition is sufficient. The feasibility, reliability and robustness of the CTM are validated not only through simulations and experiments on steels, aluminum alloys and titanium alloys, but also through a comparison with the UDM for evaluating the lower limits of fatigue strength at different SPs and confidence levels.

The LLFS is a key indicator for evaluating the fatigue performance in metallic materials, which is closely related to the sample size. Although some methods (e.g., UDM and CTM) have been established for the evaluation of LLFS in previous studies, few results are reported for the evaluation of the LLFS using the UDM and CTM based on numerous experimental data. In particular, there are no results available for the influence of the sample size on the LLFS using the UDM and CTM in the VHCF regime. This paper aims to investigate the effect of sample size on the evaluation of LLFS and provide a more reasonable and reliable way for evaluating the lower limit of high cycle and VHCF strength using the UDM or CTM. First, ultrasonic frequency fatigue tests are conducted on specimens from the TC17 titanium alloy and the welding joint of 6005A-T6 aluminum alloy, and the lower limits of the VHCF strength at 10^9^ cycles are obtained using the UDM and CTM. Then, the influence of the sample size on the lower limit of high cycle and VHCF strength, evaluated using the UDM and CTM, is studied based on the present results and the previous ones for aluminum alloys, steels and titanium alloys in the literature. The lower limits of fatigue strength evaluated using the UDM and CTM are also compared and discussed, and are compared with the minimum stress that the failure occurs at for the tested specimens during the UDM and CTM.

## 2. Materials and Methods

### 2.1. Experimental Materials

In this study, two materials are tested for the LLFS at 10^9^ cycles. One is the welding joint of 6005A-T6 aluminum alloy cut from the tractor beam of the high-speed train [21]. The Young’s modulus is 73.16 GPa and the density is 2.68 g/cm^3^ for the 6005A-T6 aluminum alloy. The other is the TC17 titanium alloy from a thick plate. Its Young’s modulus is 112 GPa and its density is 4.68 g/cm^3^ [22].

### 2.2. Fatigue Testing Methods

**Figure 1:**
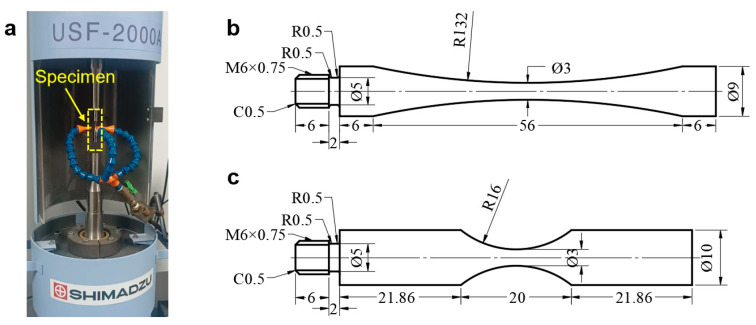
Fatigue testing machine and specimens in mm. (**a**) Ultrasonic frequency fatigue machine; (**b**) specimen of welding joint of 6005A-T6 aluminum alloy [21]; (**c**) specimen of TC17 titanium alloy.

The fatigue test is conducted on an ultrasonic frequency fatigue machine (SHIMADZU USF-2000A in Kyoto, Japan) at room temperature and in air environment (Figure 1a). The stress ratio is −1. The specimen is designed according to Refs. [21,23,24]. Figure 1b,c shows the shape and geometry of specimens from the welding joint of the 6005A-T6 aluminum alloy and the TC17 titanium alloy, respectively. The experimental section of the specimen is ground and polished before the fatigue test, and compressive cold air is used to cool the minimum cross section of the specimen during the fatigue test.

For specimens from the welding joint of the 6005A-T6 aluminum alloy, only the UDM is used to obtain the LLFS at 10^9^ cycles because the results of the CTM are available in Ref. [21]. The estimated fatigue strength at 10^9^ cycles is taken as 92.5 MPa, and the stress step is taken as 5% of the estimated fatigue strength, i.e., 4.625 MPa. Meanwhile, for the TC17 titanium alloy, both the UDM and CTM are used to obtain the LLFS at 10^9^ cycles. The fatigue tests are at first conducted under different stress levels, and then the fatigue strength at 10^9^ cycles is estimated and the step is chosen. The sample size is 15 for both the welding joint of 6005A-T6 aluminum alloy and the TC17 titanium alloy.

## 3. Lower Limit of Fatigue Strength

### 3.1. Welding Joint of 6005A-T6 Aluminum Alloy

**Figure 2:**
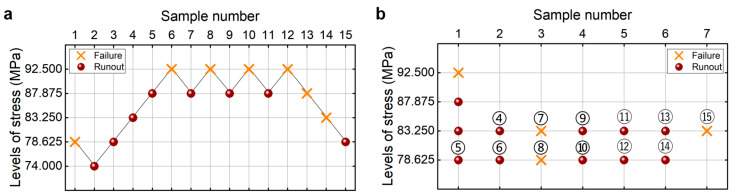
Testing results of the fatigue strength at 10^9^ cycles for the welding joint of 6005A-T6 aluminum alloy. (**a**) UDM; (**b**) CTM [21], in which the numbers ④, ⑤, ⑥, …, ⑮ denote the sample that is used for analysis.

Figure 2a shows the testing results from the UDM for the welding joint of the 6005A-T6 aluminum alloy. The detailed information is listed in Table 1. The lower limits of the fatigue strength at different SPs and 95% confidence are shown in Table 2, which are obtained through the maximum likelihood method for the UDM. For comparison, the testing results of fatigue strength at 10^9^ cycles from the CTM [21] are presented in Figure 2b and Table 2 for the same welding 6005A-T6 aluminum alloy. It is seen that the lower limits of the fatigue strength from the UDM and CTM are close to each other, at 90% and 95% SPs and 95% confidence. And these evaluated lower limits of fatigue strength are all lower than the minimum stress of 78.625 MPa that the failure occurs at for the tested specimens, indicating that both the UDM and CTM could give a safe evaluation of the fatigue strength at 10^9^ cycles for the welding joint of the 6005A-T6 aluminum alloy.

### 3.2. TC17 Titanium Alloy

**Figure 3:**
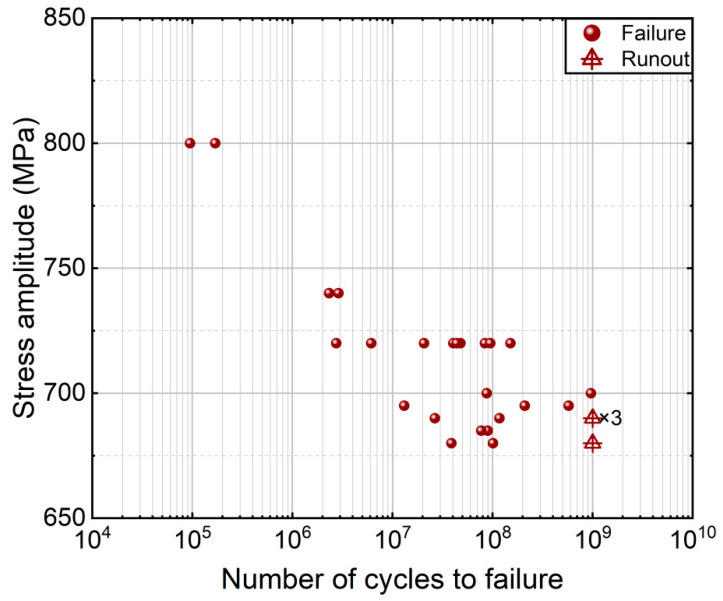
S-N data of the TC17 titanium alloy.

Figure 3 shows the testing results of the TC17 titanium alloy under different stress levels. The detailed information is listed in Table 3. It is seen from Figure 3 that the specimen of the TC17 titanium alloy could fail in the VHCF regime. The fatigue strength of the TC17 titanium alloy has a downward trend with an increasing fatigue life.

**Figure 4:**
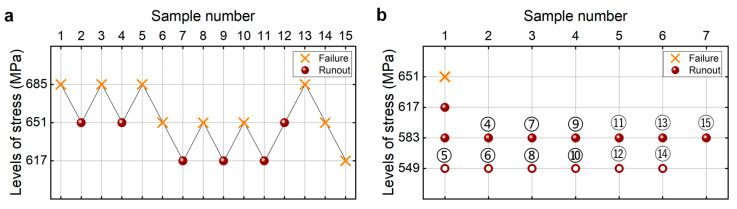
Testing results of the fatigue strength at 10^9^ cycles for the TC17 titanium alloy, in which the hollow circle denotes the hypothetical non-failure samples in the transformed four-stress-level cases. (**a**) UDM; (**b**) CTM, in which the numbers ④, ⑤, ⑥, …, ⑮ denote the sample that is used for analysis.

As is used in the literature [25], the estimated fatigue strength is taken as the average value (i.e., 685 MPa) of the tested maximum stress where the specimen does not fail after 10^9^ cycles and the tested minimum stress that the specimen fails at before 10^9^ cycles. The step stress is taken as 5% of the estimated fatigue strength, i.e., 34 MPa. The testing results of the fatigue strength at 10^9^ cycles from the CTM and UDM for the TC17 titanium alloy are presented in Figure 4. The detailed information is listed in Table 4. The lower limits of fatigue strength at different SPs and 95% confidence are shown in Table 5. It is seen that the lower limits of the fatigue strength from the UDM and CTM are close to each other at the same SP and confidence. And these evaluated lower limits of fatigue strength are all lower than the minimum stress of 617 MPa that the failure occurs at for the tested specimens, indicating that both the UDM and CTM could give a safe evaluation of the fatigue strength at 10^9^ cycles for the TC17 titanium alloy.

## 4. Effect of Sample Size on the Lower Limit of Fatigue Strength

As is well known, the estimated LLFS is closely related to the sample size. Here, the influence of the sample size on the lower limit is analyzed for high cycle strength at 10^7^ cycles and VHCF strength at 10^8^ and 10^9^ cycles based on the testing results of the welding joint of the 6005A-T6 aluminum alloy and the TC17 titanium alloy, along with the previous results for steels, aluminum alloys and titanium alloys in Refs. [17,20,21]. The fatigue strengths evaluated using the UDM and CTM at 90% and 95% SPs and 95% confidence are considered and compared. The experimental material, estimated stress level (i.e., the first stress level), step size, number of samples for analysis and the given fatigue life at which the associated fatigue strength is focused are shown in Table 6.

From the perspective of statistical analysis, more samples generally lead to a fatigue strength closer to the overall fatigue strength. To quantitatively analyze the difference between the lower limits of the fatigue strength from different sample sizes, the lower limits of fatigue strength are calculated for the first 5~14 samples in each test of the UDM or CTM, and the ones evaluated with the common size of 15 samples in the UDM are taken as reference values, i.e., the relative difference value is introduced by $\left(\frac{L_{n}}{L_{15}\;}-\;1\right)\times \;100\%$, in which *L_n_* denotes the LLFS evaluated using the first *n* samples (*n* is integer, and 5 ≤ *n* ≤ 15), and *L*_15_ denotes the LLFS evaluated using 15 samples.

For the UDM, the LLFS is calculated directly for the first 5~15 samples according to the actual testing sequence. For the CTM, it is necessary to specify the order of samples because fatigue testing under different stress levels can be conducted simultaneously in actual tests. According to the rule of the CTM [20], the samples are sorted according to the labels ④, ⑤, ⑥, …, ⑮, and they are sorted according to the actual testing sequence at the same stress level, as shown in Figure 2b. Then, the LLFS is calculated for the first 5~14 samples using the CTM. It is noted that the testing samples from the CTM at the same stress level are not always placed in the actual testing sequence in Refs. [20,21], in which the failed samples are placed at the end for the same stress level.

### 4.1. VHCF Strength of Aluminum Alloys and Welding Joints

#### 4.1.1. 6005A-T6 Aluminum Alloy at 10^9^ Cycles

**Figure 5:**
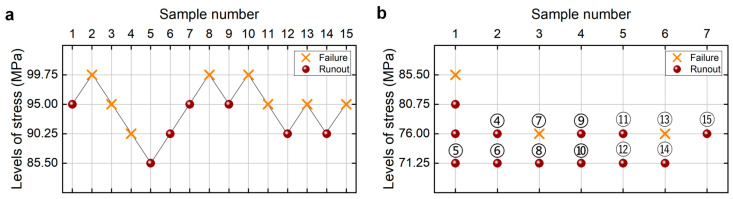
Testing results of fatigue strength at 10^9^ cycles for the 6005A-T6 aluminum alloy [20]. (**a**) UDM; (**b**) CTM, in which the numbers ④, ⑤, ⑥, …, ⑮ denote the sample that is used for analysis.

**Figure 6:**
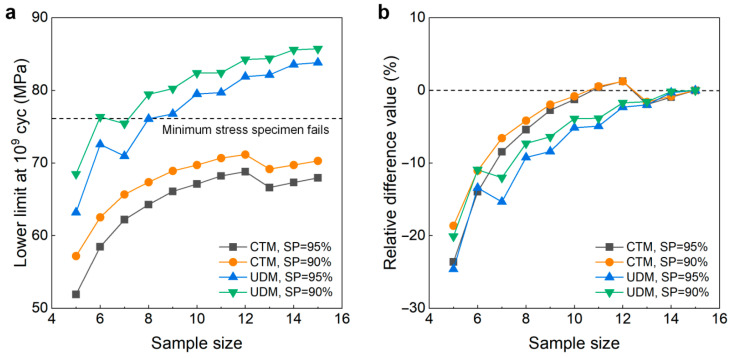
Influence of sample size on the LLFS at 10^9^ cycles for the 6005A-T6 aluminum alloy at 95% confidence. (**a**) Fatigue strength with sample size; (**b**) relative difference value with sample size.

Figure 5 shows the testing results of the fatigue strength at 10^9^ cycles for the 6005A-T6 aluminum alloy from the UDM and CTM. The influence of sample size (5 ≤ *n* ≤ 15) on the LLFS is shown in Figure 6. It is seen from Figure 6 that the sample size plays an important role in evaluating the LLFS at 90% and 95% SPs and 95% confidence. For the UDM, the evaluated LLFS generally has an increasing trend with an increasing sample size. At the sample size of 5, the evaluated LLFS is lower than the minimum stress of 76 MPa that the failure occurs at for the specimens tested using the UDM and CTM. Meanwhile, when the sample size is bigger than 7, the evaluated LLFS is higher than the minimum stress of 76 MPa at both 90% and 95% SPs and 95% confidence, indicating that the UDM gives a dangerous evaluation of the LLFS at 10^9^ cycles for the 6005A-T6 aluminum alloy. With the increase in sample size *n* (5 ≤ *n* ≤ 14), the absolute value of the relative difference value is decreased. When the sample size ranges from 10 to 14, it is within 2%.

For the CTM, the evaluated LLFS generally has an increasing trend with an increase in sample size. For the sample size ranging from 5 to 15, the evaluated LLFS is lower than the minimum stress of 76 MPa that the failure occurs at for the samples tested using the UDM and CTM. At a smaller sample size *n* (e.g., *n* < 9), the evaluated LLFS at 90% and 95% SPs and 95% confidence is obviously lower than the minimum stress of 76 MPa that the failure occurs at for the samples tested using the UDM and CTM, indicating that the CTM might underestimate the LLFS at 10^9^ cycles for the 6005A-T6 aluminum alloy. Meanwhile, when the sample size *n* is bigger (e.g., *n* ≥ 10), the difference becomes small between the evaluated LLFS and the minimum stress of 76 MPa that the failure occurs at for the samples tested using the UDM and CTM, indicating that the CTM could give a reasonable evaluation of the LLFS at 10^9^ cycles for the 6005A-T6 aluminum alloy. The absolute value of the relative difference value is usually decreased with an increase in sample size *n* (5 ≤ *n* ≤ 15), as indicated in Figure 6b. When the sample size ranges from 10 to 14, the absolute value of the relative difference value is within 6%. A comparison of the results in Figure 6 implies that, for the sample size ranging from 10 to 15, the CTM is better than the UDM for evaluating the LLFS at 10^9^ cycles for the 6005A-T6 aluminum alloy.

#### 4.1.2. Welding Joint of 6005A-T6 Aluminum Alloy at 10^9^ Cycles

**Figure 7:**
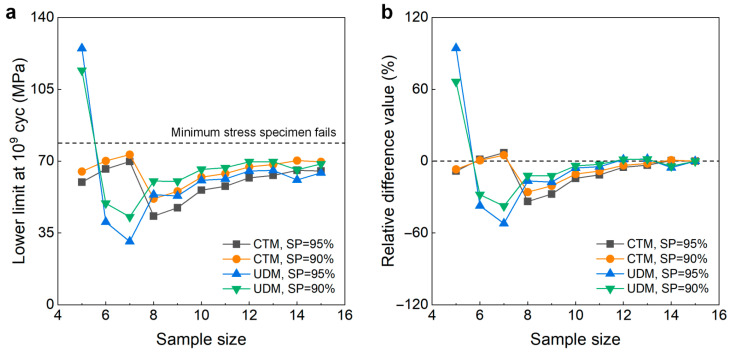
Influence of sample size on the LLFS at 10^9^ cycles for the welding joint of 6005A-T6 aluminum alloy at 95% confidence. (**a**) Fatigue strength with sample size; (**b**) relative difference value with sample size.

Figure 7 gives the influence of sample size *n* (5 ≤ *n* ≤ 15) on the LLFS at 10^9^ cycles for the welding joint of the 6005A-T6 aluminum alloy. Similarly to the 6005A-T6 aluminum alloy, the sample size plays an important role in the evaluation of the LLFS at 90% and 95% SPs and 95% confidence. For the UDM, the evaluated LLFS of five samples is much higher than the minimum stress of 78.625 MPa that the failure occurs at for the samples tested using the UDM and CTM, indicating that the UDM gives a dangerous evaluation of the LLFS at 10^9^ cycles for the welding joint of the 6005A-T6 aluminum alloy. When the sample size is bigger than 6, the evaluated LLFS generally has an increasing trend with an increasing sample size, but the evaluated lower limits of the fatigue strengths are all lower than the minimum stress of 78.625 MPa. For a sample size bigger than 9, the difference is small between the evaluated LLFS and the minimum stress of 78.625 MPa that the failure occurs at for the specimens tested using the UDM and CTM, and the absolute value of the relative difference value is less than 6%. This indicates that the UDM should give a reasonable evaluation of the LLFS at 10^9^ cycles for the welding joint of 6005A-T6 aluminum alloy when the sample size ranges from 10 to 15.

For the CTM, the evaluated lower limits of fatigue strength at 90% and 95% SPs and 95% confidence are all lower than the minimum stress of 78.625 MPa that the failure occurs at for the specimens tested using the UDM and CTM. When the sample size is bigger (e.g., *n* ≥ 10), the difference becomes small between the evaluated LLFS and the minimum stress of 78.625 MPa, and the absolute value of the relative difference value is within 15%. This indicates that, similar to the UDM, the CTM should also give a reasonable evaluation of the LLFS at 10^9^ cycles for the welding joint of the 6005A-T6 aluminum alloy for 10~15 samples. A comparison of the results in Figure 7 implies that, when the sample size ranges from 10 to 15, the evaluated lower limits of the fatigue strength at 10^9^ cycles from the UDM are mostly a little higher than those from the CTM for the welding joint of the 6005A-T6 aluminum alloy.

#### 4.1.3. 2024-T351 Aluminum Alloy at 10^7^ Cycles

**Figure 8:**
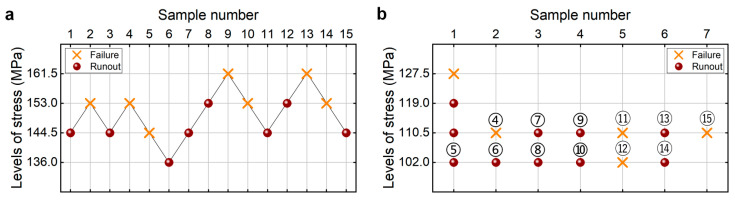
Testing results of fatigue strength at 10^7^ cycles for the 2024-T351 aluminum alloy [20]. (**a**) UDM; (**b**) CTM, in which the numbers ④, ⑤, ⑥, …, ⑮ denote the sample that is used for analysis.

**Figure 9:**
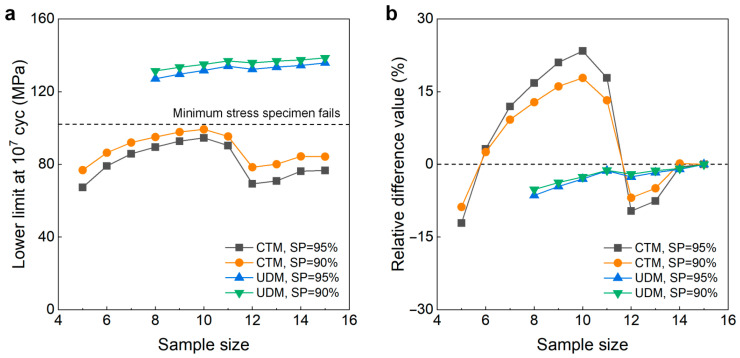
Influence of sample size on the LLFS at 10^7^ cycles for the 2024-T351 aluminum alloy at 95% confidence. (**a**) Fatigue strength with sample size; (**b**) relative difference value with sample size.

Figure 8 shows the testing results of fatigue strength at 10^7^ cycles for the 2024-T351 aluminum alloy from the UDM and CTM. The influence of the sample size *n* (5 ≤ *n* ≤ 15) on the LLFS at 10^7^ cycles is shown in Figure 9. For the UDM, the case of the results for 5~7 samples cannot be used to evaluate the LLFS through the maximum likelihood method. The evaluated lower limits of the fatigue strength using 8~15 samples are all much higher than the minimum stress of 102 MPa that the failure occurs at for the specimens tested using the UDM and CTM, which is 33.1% higher than the minimum stress of 102 MPa at 90% SP and 95% confidence and 35.9% higher than the minimum stress of 102 MPa at 95% SP and 95% confidence. This indicates that the UDM gives a very dangerous evaluation of the lower limits of the fatigue strength at 10^7^ cycles for the 2024-T351 aluminum alloy. The evaluated LLFS generally has an increasing trend with an increasing sample size. The absolute value of the relative difference value is less than 4%, indicating that the sample size of 8~15 has a small influence on the evaluated LLFS at 10^7^ cycles for the 2024-T351 aluminum alloy.

For the CTM, the evaluated lower limits of the fatigue strength at 90% and 95% SPs and 95% confidence are all lower than the minimum stress of 102 MPa that the failure occurs at for the specimens tested using the UDM and CTM. In particular, when the sample size is 12 and 13, the evaluated lower limits of the fatigue strength are relatively lower than the minimum stress of 102 MPa due to the occurrence of the failure of specimen 12 at the low stress level. Correspondingly, the variation in the absolute value of the relative difference value is bigger for the sample size of 10~14 at both 90% and 95% SPs and 95% confidence. But, overall, the CTM could give a reasonable evaluation of the LLFS at 10^7^ cycles for the 2024-T351 aluminum alloy when the sample size ranges from 10 to 15.

### 4.2. High Cycle and VHCF Strength of Steels

#### 4.2.1. G20Mn5QT Steel at 10^7^ Cycles

**Figure 10:**
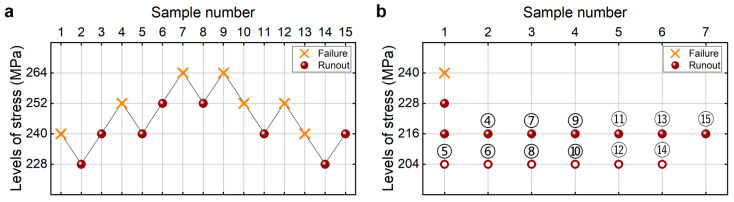
Testing results of fatigue strength at 10^7^ cycles for the G20Mn5QT steel [17], in which the hollow circle denotes the hypothetical non-failure samples in the transformed four-stress-level cases. (**a**) UDM; (**b**) CTM, in which the numbers ④, ⑤, ⑥, …, ⑮ denote the sample that is used for analysis.

**Figure 11:**
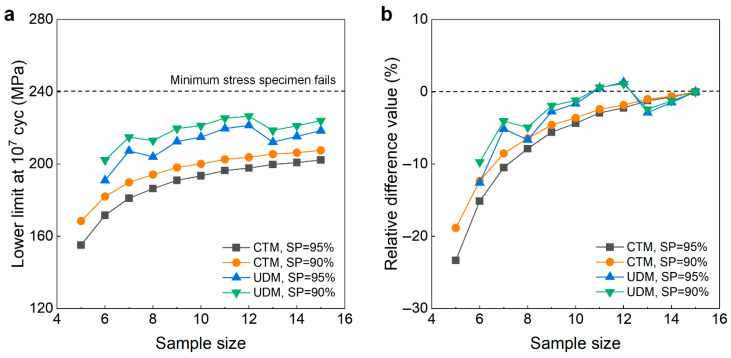
Influence of sample size on the LLFS at 10^7^ cycles for the G20Mn5QT steel at 95% confidence. (**a**) Fatigue strength with sample size; (**b**) relative difference value with sample size.

Figure 10 shows the testing results of the fatigue strength at 10^7^ cycles for the G20Mn5QT steel from the UDM and CTM. The influence of the sample size *n* (5 ≤ *n* ≤ 15) on the LLFS is shown in Figure 11. For the UDM, the case of the results for five samples cannot be used to evaluate the LLFS through the maximum likelihood method. For the CTM, the lower limits of fatigue strength are evaluated through the transformed four-stress-level cases due to the non-failure of the specimens tested at the stress level of *S*_3_ = 216 MPa [20].

It is seen in Figure 11 that the evaluated LLFS generally increases for the UDM but monotonically increases for the CTM with an increasing sample size. The evaluated lower limits of the fatigue strength at 90% and 95% SPs and 95% confidence are all lower than the minimum stress of 240 MPa that the failure occurs at for the specimens tested using the UDM and CTM. When the sample size ranges from 10 to 15, the difference often becomes small between the evaluated lower limits of the fatigue strength and the minimum stress of 240 MPa that the failure occurs at, and the absolute value of the relative difference value is also small and within 5%. This indicates that both the UDM and CTM could give a reasonable evaluation of the LLFS at 10^7^ cycles for the G20Mn5QT steel. A comparison of the results in Figure 11 implies that the evaluated LLFS at 10^7^ cycles from the UDM is higher than that from the CTM for the G20Mn5QT steel.

#### 4.2.2. As-Received 40Cr Steel at 10^7^ Cycles

**Figure 12:**
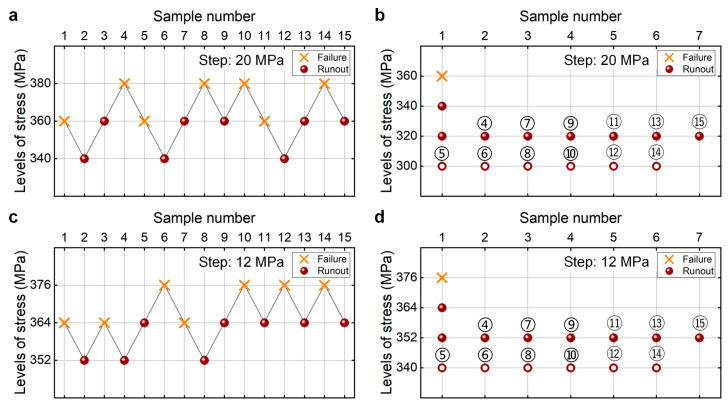
Testing results of fatigue strength at 10^7^ cycles for the as-received 40Cr steel [17,20], in which the hollow circle denotes the hypothetical non-failure samples in the transformed four-stress-level cases. (**a**,**c**) UDM with stress steps of 20 MPa and 12 MPa, respectively; (**b**,**d**) CTM with stress steps of 20 MPa and 12 MPa, respectively, in which the numbers ④, ⑤, ⑥, …, ⑮ denote the sample that is used for analysis.

**Figure 13:**
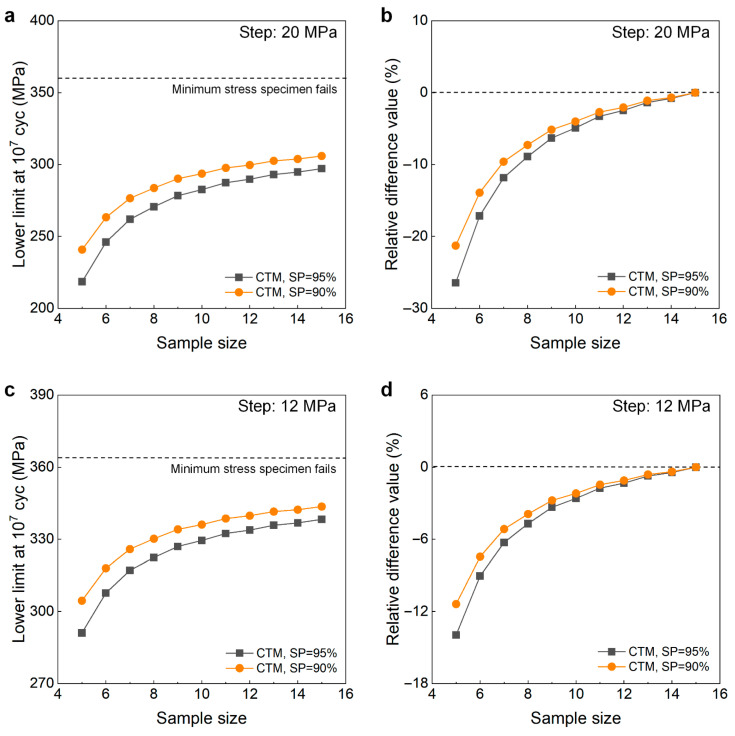
Influence of sample size on the LLFS at 10^7^ cycles for the as-received 40Cr steel at 95% confidence. (**a**) Fatigue strength with sample size for the stress step of 20 MPa; (**b**) relative difference value with sample size for the stress step of 20 MPa; (**c**) fatigue strength with sample size for the tress step of 12 MPa; (**d**) relative difference value with sample size for the stress step of 12 MPa.

Figure 12 shows the testing results of fatigue strength at 10^7^ cycles for the as-received 40Cr steel from the UDM and CTM. The influence of the sample size *n* (5 ≤ *n* ≤ 15) on the LLFS is shown in Figure 13. In Figure 12 and Figure 13, two different stress steps are considered. One is 5% (i.e., 20 MPa) of the estimated fatigue strength, and the other is 3% (i.e., 12 MPa) of the estimated fatigue strength. For the UDM, both of the results at different stress steps cannot be used to evaluate the LLFS at 10^7^ cycles through the maximum likelihood method. For the CTM, the LLFS is evaluated using the transformed four-stress-level cases due to the non-failure of the specimens tested at stress levels of *S*_3_ = 320 MPa and *S*_3_ = 352 MPa [20].

The results in Figure 13 indicate that, for both stress steps of 20 MPa and 12 MPa, the evaluated LLFS monotonically increases with an increasing sample size for the CTM, and it is lower than the minimum stress that the failure occurs at for the specimens tested using the UDM and CTM. When the sample size ranges from 10 to 15, the difference becomes small between the evaluated lower limits of the fatigue strength and the minimum stress that the failure occurs at, and the absolute value of the relative difference value is within 5%. This indicates that the CTM could give a reasonable evaluation of the LLFS at 10^7^ cycles for the as-received 40Cr steel. The results in Figure 13 also imply that the UDM is invalid for evaluating the LLFS at 10^7^ cycles for the as-received 40Cr steel due to its limitation for dealing with the testing results of three stress levels in Figure 12a,c.

#### 4.2.3. Heat-Treated 40Cr Steel at 10^8^ Cycles

**Figure 14:**
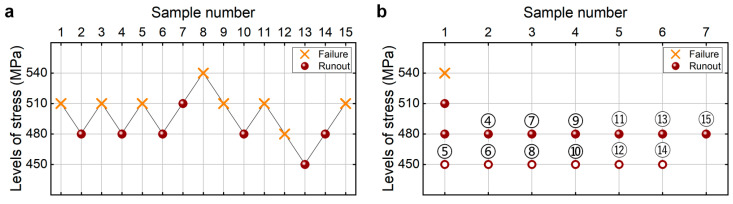
Testing results of fatigue strength at 10^8^ cycles for the heat-treated 40Cr steel [17], in which the hollow circle denotes the hypothetical non-failure samples in the transformed four-stress-level cases. (**a**) UDM; (**b**) CTM, in which the numbers ④, ⑤, ⑥, …, ⑮ denote the sample that is used for analysis.

**Figure 15:**
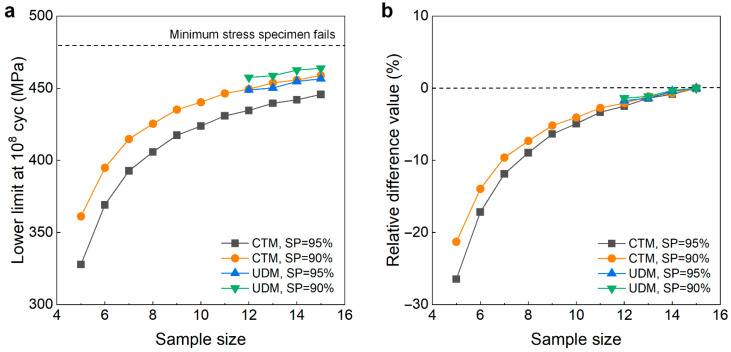
Influence of sample size on the LLFS at 10^8^ cycles for the heat-treated 40Cr steel at 95% confidence. (**a**) Fatigue strength with sample size; (**b**) relative difference value with sample size.

Figure 14 shows the testing results of fatigue strength at 10^8^ cycles for the heat-treated 40Cr steel from the UDM and CTM. The influence of the sample size *n* (5 ≤ *n* ≤ 15) on the LLFS at 10^8^ cycles is shown in Figure 15. For the UDM, the case of the results for 5~11 samples cannot be used to evaluate the LLFS through the maximum likelihood method. For the CTM, the LLFS is evaluated using the transformed four-stress-level cases due to the non-failure of specimens tested at the stress level of *S*_3_ = 480 MPa [20].

It is seen from Figure 14 and Figure 15 that the evaluated lower limits of the fatigue strength from the UDM and CTM monotonically increase with an increasing sample size, and they are both lower than the minimum stress of 480 MPa that the failure occurs at for the specimens tested using the UDM and CTM. When the sample size ranges from 10 to 15, the difference is small between the evaluated lower limits of the fatigue strength and the minimum stress of 480 MPa that the failure occurs at, and the absolute value of the relative difference value is within 5%. This indicates that both the UDM and CTM can give a reasonable evaluation of the LLFS at 10^8^ cycles for the heat-treated 40Cr steel. A comparison of the results in Figure 15 shows that the evaluated LLFS at 10^8^ cycles from the UDM is a little higher than that of the CTM for the heat-treated 40Cr steel.

### 4.3. High Cycle and VHCF Strength of Titanium Alloys

#### 4.3.1. Ti-6Al-4V Titanium Alloy at 10^7^ Cycles

**Figure 16:**
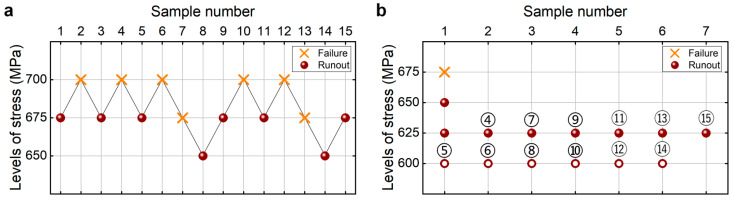
Testing results of fatigue strength at 10^7^ cycles for the Ti-6Al-4V titanium alloy [17], in which the hollow circle denotes the hypothetical non-failure samples in the transformed four-stress-level cases. (**a**) UDM; (**b**) CTM, in which the numbers ④, ⑤, ⑥, …, ⑮ denote the sample that is used for analysis.

**Figure 17:**
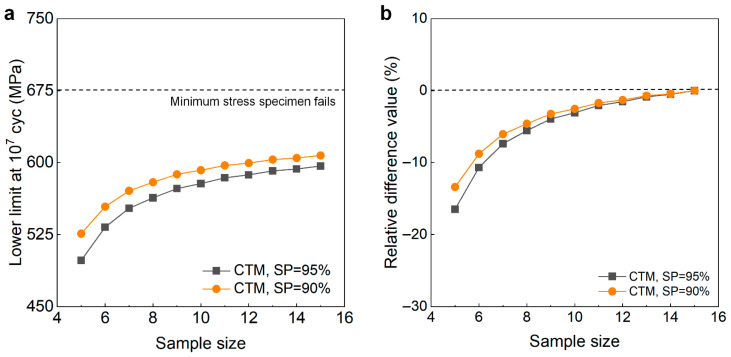
Influence of sample size on the LLFS at 10^7^ cycles for the Ti-6Al-4V titanium alloy at 95% confidence. (**a**) Fatigue strength with sample size; (**b**) relative difference value with sample size.

Figure 16 shows the testing results of the fatigue strength at 10^7^ cycles for the Ti-6Al-4V titanium alloy from the UDM and CTM. The influence of the sample size *n* (5 ≤ *n* ≤ 15) on the LLFS is shown in Figure 17. For the UDM, the tested results cannot be used to evaluate the LLFS at 10^7^ cycles for the Ti-6Al-4V titanium alloy. For the CTM, the LLFS is evaluated using the transformed four-stress-level cases due to the non-failure of specimens tested at the stress level of *S*_3_ = 625 MPa [20].

Figure 17 shows that the evaluated LLFS monotonically increases with an increasing sample size for the CTM. The evaluated lower limits of the fatigue strength are all lower than the minimum stress of 675 MPa that the failure occurs at for the specimens tested using the UDM and CTM. When the sample size ranges from 10 to 15, the difference becomes small between the evaluated lower limits of the fatigue strength and the minimum stress that the failure occurs at, and the absolute value of the relative difference value is within 4%. This indicates that the CTM could give a reasonable evaluation of the LLFS at 10^7^ cycles for the Ti-6Al-4V titanium alloy.

#### 4.3.2. TC17 Titanium Alloy at 10^9^ Cycles

**Figure 18:**
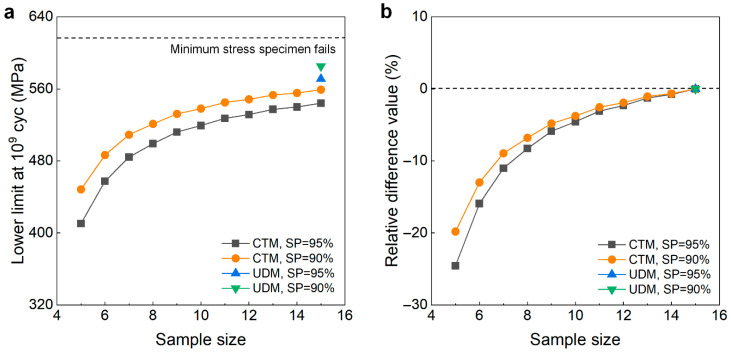
Influence of sample size on the LLFS at 10^9^ cycles for the TC17 titanium alloy. (**a**) Fatigue strength with sample size; (**b**) relative difference value with sample size.

The testing results of the fatigue strength at 10^9^ cycles for the TC17 titanium alloy from the UDM and CTM are shown in Figure 4. The influence of the sample size *n* (5 ≤ *n* ≤ 15) on the LLFS is shown in Figure 18. For the UDM, only the case of results for 15 samples can evaluate the LLFS through the maximum likelihood method. For the CTM, the LLFS is evaluated using the transformed four-stress-level cases due to the non-failure of the specimens tested at the stress level of *S*_3_ = 583 MPa [20].

It is seen from Figure 18 that the evaluated lower limits of the fatigue strength from the UDM with 15 samples are lower and close to the minimum stress of 617 MPa that the failure occurs at for the specimens tested using the UDM and CTM, indicating that the UDM could give a reasonable evaluation of the LLFS at 10^9^ cycles for the TC17 titanium alloy. Meanwhile, for the CTM, the evaluated LLFS monotonically increases with an increasing sample size, and it is lower than the minimum stress of 617 MPa that the failure occurs at. When the sample size ranges from 10 to 15, the difference becomes small between the evaluated lower limits of the fatigue strength and the minimum stress that the failure occurs at, and the absolute value of the relative difference value is within 5%. This indicates that the CTM could give a reasonable evaluation of the LLFS at 10^9^ cycles for the TC17 titanium alloy.

The results of the aluminum alloys, steels and titanium alloys show that, in some cases of testing results with three stress levels, the UDM cannot be used to evaluate the LLFS in metallic materials, while the CTM can give an evaluation of the LLFS for all the testing results. The evaluated lower limits of the fatigue strength from the UDM are generally bigger than those from the CTM at 90% and 95% SPs and 95% confidence. In some cases, the results from the UDM are very dangerous, but the ones from the CTM are all conservative.

Compared to the common size of 15 samples used in the UDM, the sample size of 10~15 can also give acceptable results for both the UDM and CTM at 90% and 95% SPs and 95% confidence. The absolute value of the relative difference value of the results of 10~14 samples compared to those of 15 samples is generally within 5%. Considering that the evaluated LLFS from the CTM is lower than the minimum stress that the failure occurs at for the specimens tested using the UDM and CTM, the LLFS at 90% SP and 95% confidence can be preferred for the fatigue strength evaluation in high cycle and VHCF regimes.

## 5. Conclusions

In this paper, the VHCF strength is first tested for the TC17 titanium alloy and the welding joint of the 6005A-T6 aluminum alloy through an ultrasonic frequency fatigue test. Then, the influence of the sample size on the LLFS using the UDM and CTM is investigated for high cycle and VHCF strength (10^7^, 10^8^ and 10^9^ cycles) based on the present results and the ones in the literature. The novel results are as follows.

(1) The evaluated LLFS is closely related to the sample size for both the UDM and CTM, which often has an increasing trend with the increase in sample size *n* (5 ≤ *n* ≤ 15). Generally, the increasing trend is relatively slow when the sample size *n* ≥ 10. The absolute value of the relative difference value of the results of 10~14 samples compared to those of 15 samples is generally within 5% at 90% and 95% SPs and 95% confidence, indicating that 10~14 samples can give acceptable results compared to 15 samples for both the UDM and CTM.
(2) The evaluated lower limits of the fatigue strength from the UDM are generally higher than those from the CTM. In some cases, the results from the UDM are very dangerous. For example, the evaluated LLFS at 95% SP and 95% confidence for 15 samples is 10.3% higher than the minimum stress that the failure occurs at for the specimens tested using the UDM and CTM for the 6005A-T6 aluminum alloy. The evaluated lower limits of the fatigue strength are all conservative for the CTM, and they are generally close to the minimum stress that the failure occurs at when the sample size ranges from 10 to 15.
(3) The CTM deals with all the testing results, while the UDM fails to evaluate the LLFS in some cases. The CTM could give a more reasonable evaluation of fatigue strength because more specimens are tested under lower stress levels for the same sample size. Ten samples and the LLFS at 90% SP and 95% confidence can be a better choice when economic and time costs are considered.

## Figures and Tables

**Table 1:** Testing results for the welding joint of 6005A-T6 aluminum alloy in Figure 2a.

Specimen Number	*σ*max/MPa	Cycles	Failure or Runout
1	78.625	9.55 × 10^8^	Failure
2	74	1.00 × 10^9^	Runout
3	78.625	1.00 × 10^9^	Runout
4	83.25	1.00 × 10^9^	Runout
5	87.875	1.00 × 10^9^	Runout
6	92.5	9.67 × 10^8^	Failure
7	87.875	1.00 × 10^9^	Runout
8	92.5	1.81 × 10^8^	Failure
9	87.875	1.00 × 10^9^	Runout
10	92.5	1.91 × 10^8^	Failure
11	87.875	1.00 × 10^9^	Runout
12	92.5	5.69 × 10^8^	Failure
13	87.875	7.18 × 10^8^	Failure
14	83.25	2.66 × 10^8^	Failure
15	78.625	1.00 × 10^9^	Runout

**Table 2:** LLFS at 10^9^ cycles for the welding joint of 6005A-T6 aluminum alloy at different SPs and 95% confidence.

Testing Method	Lower Limit at 90% SP/MPa	Lower Limit at 95% SP/MPa
UDM	68.64	64.32
CTM [21]	69.68	65.21

**Table 3:** Testing results for the TC17 titanium alloy in Figure 3.

Specimen Number	*σ*max/MPa	Cycles	Failure or Runout
1	800	1.7 × 10^5^	Failure
2	800	9.5 × 10^4^	Failure
3	740	2.3 × 10^6^	Failure
4	740	2.9 × 10^6^	Failure
5	720	2.1 × 10^7^	Failure
6	720	1.5 × 10^8^	Failure
7	720	4.8 × 10^7^	Failure
8	720	4.1 × 10^7^	Failure
9	720	6.1 × 10^6^	Failure
10	720	8.4 × 10^7^	Failure
11	720	9.5 × 10^7^	Failure
12	720	4.4 × 10^7^	Failure
13	720	2.7 × 10^6^	Failure
14	700	8.8 × 10^7^	Failure
15	700	9.6 × 10^8^	Failure
16	695	5.8 × 10^8^	Failure
17	695	2.1 × 10^8^	Failure
18	695	1.3 × 10^7^	Failure
19	690	1.0 × 10^9^	Runout
20	690	1.0 × 10^9^	Runout
21	690	1.2 × 10^8^	Failure
22	690	1.0 × 10^9^	Runout
23	690	2.7 × 10^7^	Failure
24	685	8.9 × 10^7^	Failure
25	685	7.7 × 10^7^	Failure
26	680	1.0 × 10^9^	Runout
27	680	3.9 × 10^7^	Failure
28	680	1.0 × 10^8^	Failure

**Table 4:** Testing results for the TC17 titanium alloy from UDM and CTM in Figure 4.

Method	Specimen Number	*σ*_max_/MPa	Cycles	Failure or Runout
UDM	1	685	2.6 × 10^7^	Failure
	2	651	1.0 × 10^9^	Runout
	3	685	9.2 × 10^7^	Failure
	4	651	1.0 × 10^9^	Runout
	5	685	1.0 × 10^8^	Failure
	6	651	1.5 × 10^8^	Failure
	7	617	1.0 × 10^9^	Runout
	8	651	4.2 × 10^7^	Failure
	9	617	1.0 × 10^9^	Runout
	10	651	1.8 × 10^8^	Failure
	11	617	1.0 × 10^9^	Runout
	12	651	1.0 × 10^9^	Runout
	13	685	1.1 × 10^8^	Failure
	14	651	9.5 × 10^8^	Failure
	15	617	4.3 × 10^7^	Failure
CTM	16	651	1.2 × 10^8^	Failure
	17	617	1.0 × 10^9^	Runout
	18	583	1.0 × 10^9^	Runout
	19	583	1.0 × 10^9^	Runout
	20	583	1.0 × 10^9^	Runout
	21	583	1.0 × 10^9^	Runout
	22	583	1.0 × 10^9^	Runout
	23	583	1.0 × 10^9^	Runout
	24	583	1.0 × 10^9^	Runout

**Table 5:** LLFS at 10^9^ cycles for the TC17 titanium alloy at different SPs and 95% confidence.

Testing Method	Lower Limit at 90% SP/MPa	Lower Limit at 95% SP/MPa
UDM	585.1	571.1
CTM	559.1	544.1

**Table 6:** Summary information of research plan for the effect of sample size.

Materials	Testing Method	Estimated Stress Level/MPa	Step Size/MPa	Number of Samples	Given Fatigue Life/cyc
TC17 titanium alloy	UDM	685	34	5~15	1 × 10^9^
	CTM				
Welding joint of 6005A-T6 aluminum alloy	UDM	92.5	4.625		1 × 10^9^
	CTM				
6005A-T6 aluminum alloy	UDM	95	4.75		1 × 10^9^
	CTM				
2024-T351 aluminum alloy	UDM	170	8.5		1 × 10^7^
	CTM				
G20Mn5QT steel	UDM	240	12		1 × 10^7^
	CTM				
As-received 40Cr steel	UDM	400	20		1 × 10^7^
	CTM				
	UDM	400	12		1 × 10^7^
	CTM				
Heat-treated 40Cr steel	UDM	600	30		1 × 10^8^
	CTM				
Ti-6Al-4V titanium alloy	UDM	500	25		1 × 10^7^
	CTM				

## Data Availability

The original contributions presented in this study are included in the article. Further inquiries can be directed to the corresponding authors.

## Abbreviations

The following abbreviations are used in this manuscript:

UDM	Up-and-down method
CTM	Continuous testing method
